# Hemispheric disparity theory: a dual-system framework for consciousness

**DOI:** 10.3389/fpsyg.2026.1727527

**Published:** 2026-05-29

**Authors:** Mohammad Wafa

**Affiliations:** 1 Neuroscience and Trauma, Queen Mary University of London, London, United Kingdom; 2 Croydon Health Services NHS Trust, London, United Kingdom; 3 Princess Alexandra Hospital NHS Trust, Harlow, United Kingdom

**Keywords:** alien hand, artificial intelligence, consciousness, disconnection syndrome, hemispheric asymetry, hemispheric specialization, laterality, split brain syndrome

## Abstract

The Hemispheric Disparity Theory conceptualizes one facet of consciousness, defined as the “conscious-mind” experience. Rather than a unified agent, conscious experience arises from the dynamic interplay between a left hemisphere specialized in order and abstraction through dynamic temporal modeling, and a right hemisphere specialized in contextual integration, coherence, and model updating through attentional networks. Drawing on evidence from diverse classical neuropsychological and modern neuroimaging studies, the hemispheric disparity model proposes, in Part I, how the unified conscious experience emerges from two structurally distinct cerebral hemispheres. In Part II, a demonstration of how information processing progresses to a remarkable form of asymmetry through a special structural arrangement that begins at the lower levels of cortical hierarchies. Finally, in Part III, the article proposes how the disparate hemispheric system operates to generate cumulative concept development unique to the conscious-mind. Simple mathematical concepts are used to illustrate this. This model offers potential for further development on multiple levels, including clinical, philosophical, and modern artificial learning systems.

## Defining consciousness and its context

Consciousness is a recent English polysemous term; its first recorded use dates to the 17th century, with variable medical, social, philosophical, and cognitive meanings (Barfield, 2002). This polysemic nature of the word reflects the growing interest in the subjective human experience over the last few centuries (Box 1).

Box 1 **What’s new in hemispheric disparity theory**

• In an analogy to ocular disparity, Hemispheric Disparity Theory introduces how each cerebral hemisphere cortical system generates unique conscious experience through the overlapping conscious view of reality.

• Hemispheric specialization permitted different types of information processing in each hemisphere analyzing the same reality content.

• Hemispheric specialization serves as a necessary foundation for different information processing types and different behavioral responses and is not merely for energy saving purpose within the large brain size.

• This disparate hemispheric processing can be recursive allowing for cumulative construction of concepts unique to the conscious-mind mental experience.

• Hemispheric disparity opens a potential for further development of different clinical disease models, philosophical understanding of consciousness as well as artificial learning and intelligence.

Even within the realms of cognitive neuroscience and philosophy of mind, it remains challenging to find a unified understanding of consciousness. Theories on philosophy of the mind ideas generally agree on two defining components of the conscious mind: the experiencing self and the experienced external world. “the subjective experience of the mind” as for Chalmers (1997), “capturing the self in the act of knowing,” as described by Damasio and Dolan (1999); or “The capacity of an organism to feel, subjectively experience, and integrate sensory input into unified, behaviorally relevant representations, including an awareness of the self in space and time” as for Feinberg and Mallatt (2025). For all, it is agreed that human conscious experience involves two features: “acquiring knowledge or experience” and the “agent,” the sense of “self-awareness” of that experience.

Cognitive neuroscience, on the other hand, starts with consciousness, from where it can be tested and modeled. The first serious attempts began with the neural correlates of consciousness (Crick and Koch, 1990) and later evolved over time into multiple models of consciousness that have well impacted the field (Seth and Bayne, 2022).

For some models, low-level phenomenal representations may be sufficient to establish conscious experience, even when it is non-reportable, as in the micro-consciousness and neural stance models (Zeki, 2007; Lamme, 2006).

For other theories, a second, higher-level representation of those lower-level perceptions is needed to account for conscious experience. Higher-order Theories (HOTs) (Lau and Rosenthal, 2011; Fleming, 2020; Lau, 2019), Predictive processing theories (Hohwy and Seth, 2020; Clark, 2013), as well as the Global Neuronal Workspace Theories (GWTs) (Baars, 2005; Dehaene and Changeux, 2011; Bor and Seth, 2012). Integrated information theory (IIT) posits consciousness as a fundamental property of any complex integrated information system, not restricted to human consciousness or even to biological systems (Tononi et al., 2016; Seth et al., 2011).

Consciousness, as an overarching concept, encompasses multiple facets, all referred to as “consciousness” across diverse literature without further qualification of the term. In a way to avoid confusion, the hemispheric disparity model attempts to explain only a particular facet of consciousness, which is described here as the “conscious-mind.” Conscious-mind is defined as the capacity to appreciate the personal subjective experience through a dynamic temporal context. This “mind” qualifier is intended to focus the discussion solely on the part of the consciousness process mediated by higher-order cortical systems, distinct from phenomenal or lower-level cortical and subcortical components.

As will be demonstrated here, this conscious-mind process is proposed as an emergent property of a disparate, highly specialized hemispheric process which can explain a wide variety of mental experiences.

This article is divided into three parts. First, a demonstration of how the conscious-mind functions through integrated lateralized networks in both hemispheres, and an exploration of the unified conscious experience. Second, the concept of hemispheric disparity will be examined in detail: how the two hemispheres operate on the same reality resources but progress through information processing in different directions, thereby generating a disparate yet integrated view of the world. And finally, on the third part, demonstrating examples of how such a proposed framework generates such cumulative knowledge acquisition unique to the conscious-mind.

## **P**art 1: how the two cerebral hemispheres generate a unique conscious-mind experience

Nervous system asymmetry is a feature of almost all bilaterians; however, the human brain in particular exhibits the most pronounced form of hemispheric specialisation and functional asymmetry (Ocklenburg and Güntürkün, 2024). The first groundbreaking description of the lateralization of the language function by Dax and Broca sparked the subsequent discoveries of the high functional asymmetry of both hemispheres through lesion studies (Broca, 1861; Buckingham, 2006; Dax, 1865). Interestingly, comparative lesion studies have not demonstrated a comparable strong lateralization dependency in other primates for functions such as visuospatial navigation and vocalization (Oleksiak et al., 2011; Ocklenburg et al., 2013). This fact reflects the magnitude of specialization and laterality within the human brain.

Given that the two cerebral hemispheres are functionally distinct and specialized in the human brain, it is interesting to explore how each hemisphere colors the conscious experience and how these contributions are integrated into a unified experience.

Each hemisphere is equipped with neural substrates sufficient to generate consciousness under the existing model of consciousness. The overall arrangement of lower– and higher–order systems is inherent in each hemisphere: subcortical regions, an increasing hierarchy of cortical areas, and networks organized in both domain-specific and higher-order, domain-general networks. This should not be surprising given that patients with significant damage to one hemisphere can retain consciousness, attention, and aspects of cognitive function. Additionally, Wada testing demonstrates that we can anaesthetize a whole hemisphere while preserving a form of conscious experience (Wada and Rasmussen, 2007). Patients with hemispherectomies also experience a unified full conscious experience (Kliemann et al., 2019).

Classical split-brain studies provide valuable insights into the integration required between the two hemispheres and their impact on conscious experience, behavior, and interaction with the environment. Patients who undergo complete callosotomy, a procedure that separates the two hemispheres’ cortical systems, generally do not face significant functional disabilities. In these classical experiments, it was shown that each hemisphere can independently engage in coherent conscious activity (Gazzaniga et al., 1962; Gazzaniga, 2000).

**Table 1:** 

Postulate 1: Each cerebral hemisphere is structurally sufficient to generate a form of conscious experience. Within each hemisphere there are multiple parallel integrated cortical systems arranged in a hierarchy of lower-order and higher-order cortical networks.

### Split-brain unsplit mind

In a split-brain, while basic sensory and motor functions are completely split, strikingly, these patients appear to retain a single, seemingly unified conscious access and experience until it is challenged in a specific experimental environment (Gazzaniga, 2000; Sperry, 1968; Pinto et al., 2017). Significant attention has been paid to the differences between the brain’s two hemispheres, but it is also important to consider their shared aspects and capabilities.

There are key behavioral and neuropsychological observations on these shared capabilities:

1. Lower-level sensorimotor networks on each side carry a basic bilateral representation:

- Each hemisphere is equipped with basic perceptual representations of the self and the world. Basic subcortical awareness systems operate on somatosensory, auditory, and visual information. Split-brain patients consistently showed behavior reflecting the ability to process the self and the world as a whole (Trevarthen, 1968; Schneider, 1969; Rosenzweig, 1951).
- A disconnected hemisphere can control and coordinate both arms, but control of the dominant hand is lateralized. This is supposed to be carried through the ipsilateral descending motor pathways (Gazzaniga, 2000; Gazzaniga and Sperry, 1967).
- Each hemisphere can direct visual attention to the right or left visual hemifields (Holtzman et al., 1981). Each hemisphere can direct overt attention despite the lack of integration across different visual hemifields.
  2. Lateralized higher control networks on each side act unsplit:
- Spatial attention cannot be divided in patients with a split-brain. In experimental settings, patients were unable to perform separate spatial attention tasks with each hemisphere simultaneously (Kingstone and Gazzaniga, 1995; Corballis, 1995). This implies that each control network within each hemisphere is acting with some independence rather than within one half of the environment or the body. This contrasts with their remarkable ability to perform simple motor tasks with each hand simultaneously, with minimal interference. People with an intact corpus callosum find it challenging to draw a rectangle with one hand while drawing a circle with the other hand simultaneously. Split-brain patients can perform this without difficulty. These motor tasks are less demanding and are thought to operate within action-mode networks and premotor regions in each hemisphere (Dosenbach et al., 2025). This lower-level split behavior is not reflected in the higher-level network function.
- Attentional resources and capacity are shared between the two hemispheres. For example, engagement in a demanding task in one hemisphere can affect the performance of the other, disconnected hemisphere (Holtzman and Gazzaniga, 1982; Ivry et al., 1998). This may be because each high-level control network uses the same subcortical body resources to regulate attentional capacity.
- Provided language information is accessible to the lateralized language networks; language comprehension and expression did not exhibit any splitting phenomenon in split-brain patients. Unlike in patients with right hemispheric damage, reading neglect is not a problem in these patients, implying awareness of the involved hemisphere (usually left) and of the whole environment and the reading task at hand.

The hemispheric disparity model proposes that higher lateralized functional networks in each hemisphere operate upon a complete version of reality, “the self and external world.” This was even achieved in split-brain patients, owing to the presence of lower-level representations of the self and the external world in each hemisphere.

Modern neuroimaging studies further support the domain-general nature of higher-order brain networks as outlined next.

### Functional asymmetry across higher function networks

Modern functional neuroimaging reshaped our view of the brain into distinct functional networks (Yeo et al., 2011; Power et al., 2011). Some networks show relatively strong symmetry, such as the somatomotor, visual, default mode and action mode networks (Dosenbach et al., 2025; Yeo et al., 2011). Other networks, such as language, attentional, and control networks, exhibit strong asymmetry. Sensorimotor networks’ right–left integration is callosal dependent, unlike asymmetrical networks, which are connected by less myelinated callosal commissural networks that may even maintain such asymmetry through inhibitory connections (Schulte and Müller-Oehring, 2010; Turgut et al., 2023; Jäncke and Steinmetz, 2003; Josse et al., 2008; Karolis et al., 2019).

Asymmetrical lateralized networks are known for their domain-general nature; their engagement and function are not restricted to a single sensory modality or side. This network processes language-related content, whether auditory, visual, or tactile, and whether presented to the right or left hemispheres (Fedorenko and Thompson-Schill, 2014; Hickok and Poeppel, 2004). On the right hemisphere, the ventral frontoparietal network is strongly lateralized and is part of the ventral attention network. This ventral attentional network responds to bottom-up stimuli regardless of their side and sensory modality (Corbetta et al., 2008).

The dorsal attentional network and frontoparietal control networks usually show bilateral activation; however, a distinct asymmetry is observed (Habas et al., 2009). The left frontoparietal networks have more intrahemispheric than bilateral connectivity, are linked to the default mode and language networks, and, unsurprisingly, are linked to conscious report (Liu et al., 2023; Wang et al., 2014). The right frontoparietal network maintains relatively better connectivity with the right attentional network system, besides maintaining a bilateral connectivity pattern compared to the left frontoparietal networks (Wang et al., 2014; Gotts et al., 2013).

Interestingly, recent evidence suggests that the superior frontoparietal networks on both sides exhibit an evolved bilateral representation of space. These networks are involved in top-down attentional and spatial control, working memory, as well as task switching and cognitive flexibility (Tamber-Rosenau et al., 2018; Majerus et al., 2018). This evolutionary adaptation in the human brain contrasts with the strictly contralateral representation in each hemisphere of primates (Patel et al., 2015).

Furthermore, at the global interhemispheric level, increased laterality within these higher-level networks was associated with fluid intelligence (Liang et al., 2025; Jin et al., 2024). That is to say the more independent each hemisphere is, the more it mediates higher-order functions.

As those higher-functioning networks operate at an integral (whole-body, whole-environment) domain-general level (not limited to a sensory modality), independent of strong callosal connectivity, their functioning was not compromised in split-brain patients. Also, as these networks operate in real-time within the same reality repertoire, it is fair to conclude that each hemisphere’s higher functioning is overlaid on top of its fellow hemisphere rather than each side of any given higher-network domain contributes to half or a split part of an overall function.

**Table 2:** 

Postulate 2: Within each hemisphere, a fundamental kind of bilateral mapping exists, despite primary sensorimotor mapping being unilateral and integrated via the corpus callosum. Utilizing this fundamental bilateral mapping, higher-order lateralized networks can function within an integral, unsplit representation of reality. This higher functioning was not compromised following callosal disconnection implying a considerable level of independence.

### The monolithic mind: is it a merged, overlaid function, or both?

Within the highly lateralized functions of the brain, both sides operate simultaneously on the same perceptual targets or action plan, each from a different perspective; thus, the description of overlaid functions better captures this higher-level process than the simple merging of sensory maps or the integrated bimanual function. Extending this overlaid perspective into the consciousness domain, the conscious-mind experience defined earlier is a dual-hemispheric overlaid process.

1. Appreciate the personal subjective experience
2. Through a dynamic temporal context.

These two defining features are proposed as separate lateralized functions, with the right hemisphere specializing in an integrated sense of self. In contrast, the left hemisphere overlays a temporal dynamic to this subjective experience. Further discussion on this is in Parts II and III of this article.

Hemispheric disparity here provides a higher-order model of consciousness (in particular, the conscious-mind), similar to HOTs, predictive processing, and GWT theories. However, the two defining features proposed here are products of two disparate hemispheric specializations, each lateralizing to a separate hemisphere. Hemispheric disparity theory suggests that the conscious-mind arises from a “higher-higher” dual-process mechanism rather than the “higher-lower” representations described in other models of consciousness. To put it in simpler words, the conscious-mind is the reflection of a higher process on another higher process, not a lower one.

**Table 3:** 

Postulate 3: Only through two disparate hemispheric representation of the self, the “conscious-mind” quality emerges.

There is lower-level hierarchical processing within the brain that may mediate certain aspects of phenomenal consciousness. Subcortical systems and basic sensorimotor processing mediate a form of consciousness that is comparatively widespread among vertebrates, mammals, and primates. Part of these processes can be split with corpus callosum commissurotomy in humans. There is, however, a different trait in conscious experience, defined here as the conscious-mind process, which is parallel, highly lateralized, and not callosal-dependent.

Having two separate systems mediating a conscious-mind experience might seem counterintuitive, given the unified conscious experience we all encounter. Here again, split-brain surgeries provided an excellent, unique source of information for exploring this conscious unity, and this is addressed next.

## The apparent unity explains the split-brain paradox

The paradoxical nature of split-brain consciousness stems from the following logical argument (Schechter, 2018).

1. Each person has one mind that is a product of one integrated brain,
2. Split-brain hemispheres, resulting from commissurotomy, produce two dissociated conscious experiences.
3. Observed and reported Conscious experience in split-brain patients is largely unified.

Given that the three statements are contradictory, one of the above statements must be false. Roger Sperry sought to confirm that split-brain patients have two separate conscious experiences, thereby falsifying the third statement (Sperry, 1968; Sperry, 1984; Sperry, 1966). Other attempts have argued that there is no dual-conscious experience, offering different explanations, as in Gazzaniga (2000), Wolford et al. (2000), and Pinto et al. (2017). As will be outlined in this section, hemispheric disparity tries to make the argument that the unity of the conscious-mind is only apparent and the corpus callosum is not essential in maintaining such unity. This is a rejection of the first statement in the argument above.

The overlaid nature of the conscious-mind within the hemispheric disparity model differs from the above explanations in different key points. It is a form of dual-conscious expertise, similar to Sperry’s classical views, but in an overlaid rather than split manner and only at the higher level, thus it appears integrated and unified.

Gazzaniga tried to avoid the conflicting dual conscious experience by centralizing consciousness into one hemisphere, the left interpreter hemisphere. This view clearly underestimates the right hemisphere’s equal contribution to overall brain function; recognition, the integrated sense of self, and attentional control mechanisms are difficult to exclude from the model of conscious experience. It is also biologically counterintuitive, as equal energy expenditure by the two hemispheres would be unjustified.

The hemispheric disparity model conforms with the split-perception, unified consciousness model proposed by Pinto. Pinto’s model posited that unified conscious experience is mediated by functional synchrony or by subcortical mechanisms. Electrophysiological evidence does not support functional synchrony after callosotomy (Berlucchi, 1966; Avvenuti et al., 2020). Subcortical mechanisms, although they exist and operate in a unified fashion, leave the question of why two separate cortical systems do not show contradictory behavior unanswered (Corballis et al., 2018).

**Figure 1:**
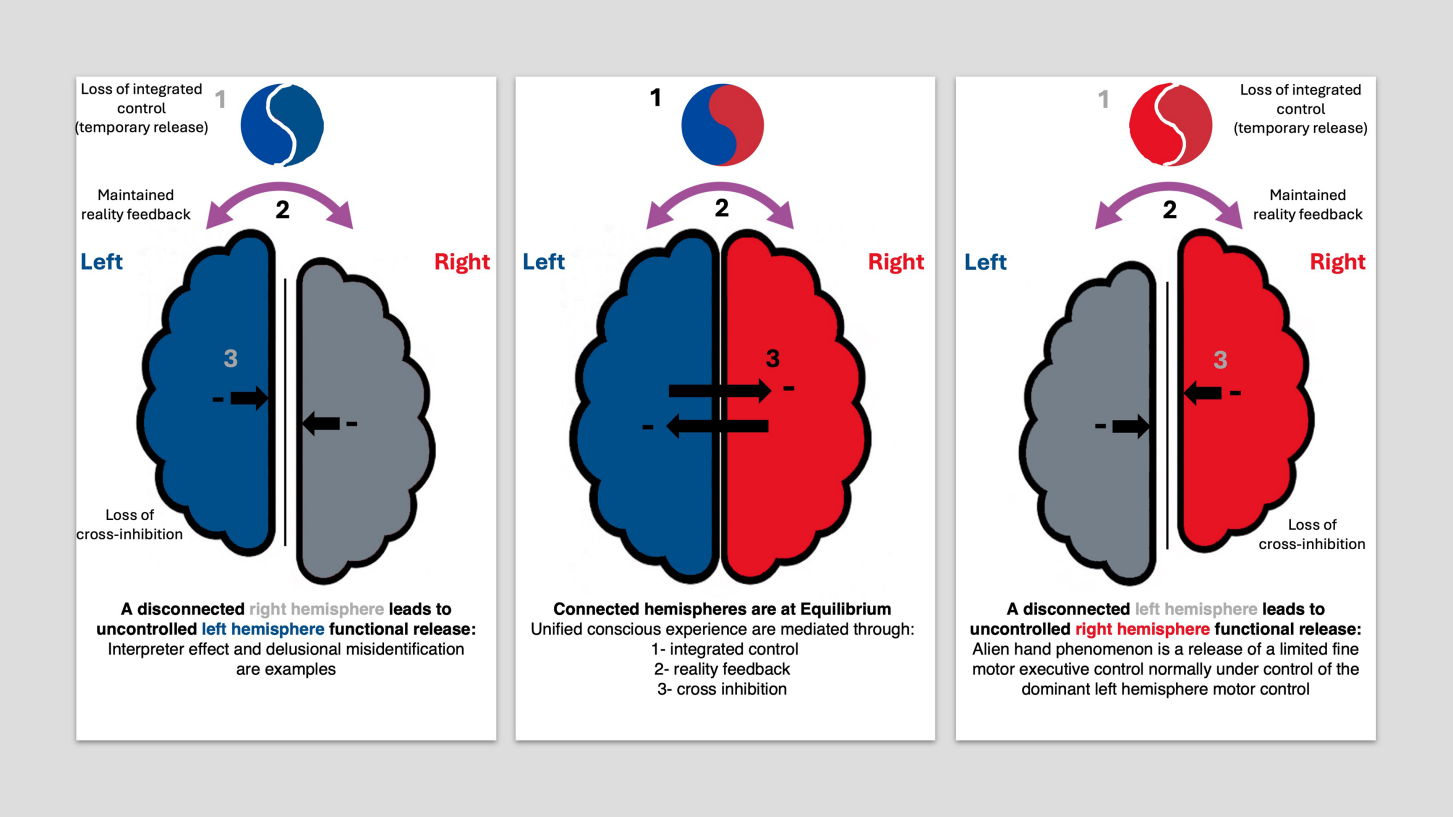
Hemispheric release effect. The three proposed mechanisms maintaining conscious unity are outlined, the reality feedback remains uninterrupted which supports the unity of experience even in split-brain patients. Temporarily integrated control is interrupted releasing contradictory behavior following surgeries. Cross inhibition remains persistently interrupted but did not contribute to a major disability to split-brain patients.

Few key mechanisms can mediate such apparent unity of the conscious-mind: integrated control, cross-inhibition and reality feedback (Figure 1).

### Integrated control: the interpreter and the alien

Integrated control means that each hemisphere is responsible for a higher-level control function that does not overlap with the other hemisphere’s control domain. Briefly, the right hemisphere controls the updating of reality models; the precision, and the details of perception of the body and the external world. The left hemisphere engages in behavioral interaction that ultimately serves to re-update the reality model perceived by the right hemisphere.

A general function of the prefrontal cortex, “action cortex,” is to maintain control over lower-level actions. Once this control is lost, a form of release syndrome occurs (Fuster, 2013). Release syndrome is defined as an unrestrained activity of a lower brain center resulting from damage or suppression of a higher control center. Following callosal disconnection, each hemisphere is released from the other hemisphere’s prefrontal control. This is a special situation of frontal release; a cross-release phenomenon.

Attention and motor planning are distinct action functions of the prefrontal cortex, lateralized to different hemispheres. Attention is the active process of inferring the level of uncertainty or precision (belief) about the external world or the body (Feldman and Friston, 2010; Parr and Friston, 2019). At the same time, motor actions select new sensory data to test hypotheses about these causes (Friston et al., 2012).

During the early post-surgical period, split-brain patients exhibited variable clinical syndromes reflecting the cross-release effect, such as the alien hand phenomenon—typically affecting the non-dominant hand—and delusional interpretations, typically associated with the left hemisphere.

#### Callosal alien hand phenomenon

The alien hand is a form of complex, unwilled motor act that represents a release from the dominant executive motor system. For fine motor tasks, the non-dominant (usually the right) hemisphere is released from left-hemisphere control, leading to the temporary emergence of the alien hand phenomenon in some patients during the early post-surgical period. This release effect occurs on multiple hierarchies. At the lowest level, patients automatically manipulate objects within reach without inhibitory, purposeful control (Mori and Yamadori, 1982; Rj, 1977). At another level, the alien hand mirrors or interferes with the movements of its fellow arm, likely via ipsilateral motor pathways (Gazzaniga et al., 1962; Fisher, 1963). At a higher level, conflicting behavior can be observed beyond hand control, for example, in tasks involving bilateral actions such as walking or dressing. Patients can be observed engaging in and disengaging from acts, repeatedly dressing before undressing, and remaining motionless despite their intention to move forward (Gazzaniga et al., 1962; Akelaitis, 1945).

#### The interpreter phenomenon

The right hemisphere attentional resources maintain an active process of constructing reality through both bottom-up and top-down processes (Corbetta et al., 2008; Corbetta et al., 1993). Perceptual completion and different tricks of visual illusions have been demonstrated to be right-hemisphere driven (Corballis, 2003; Weidner et al., 2010; Inui et al., 2000).

The left hemisphere suffers a cross-release effect once disconnected from these attentional resources. At the lowest level of the cross-release, it fails to create a coherent, complete model of reality, resulting in the classically described neglect syndromes, which can involve either the body, space, or objects.

At the next level of cross-hemispheric release, a dissociation does occur between integrated cognitive functions, such as that between the recognition function of the right hemisphere and the language left hemisphere resources. Also, in making moral judgments, whether based on intentionality (right) or verbal moral vignettes (left) (Steckler et al., 2017).

At the highest amodal level, judgment dissociates from reality testing and results in various delusional disorders, such as delusional disorders that involve the self or others; Capgras and Fregoli syndrome. The Capgras syndrome is a delusional disorder where a person falsely believes that a familiar person, or even an object or place, has been replaced by an imposter. In comparison, the Fregoli syndrome, also known as Fregoli delusion, is a rare psychological condition in which a person believes that a familiar person is disguised as different, unrelated individuals they encounter. The “interpreter” observation made by Gazzaniga is just another example of such dissociation. Michael Gazzaniga’s “interpreter” describes the left hemisphere’s tendency to create dynamic narratives and logical explanations. In normal individuals, this lack of perceptual information disrupts judgment and behavior until reality is rechecked. This is part of the right hemisphere’s inferior frontoparietal networks, as will be discussed later, a circuit breaker that directs attentional resources and updates the mental models available for conscious access (Corbetta et al., 2008).

Each control system in the right and left hemispheres maintains a separate line of work: one that internalizes reality through attentional modulation and another that manipulates reality through motor actions. The two lines of work do not oppose one another; subsequently, even if both hemispheres become disconnected, there is no apparent conflict.

### Cross inhibition

Homotopic callosal fibers are both excitatory and inhibitory. The heteromodal anterior and posterior association areas have stronger within-hemisphere than interhemispheric connectivity (Hervé et al., 2013). These areas also receive less myelinated fibers, which mostly exert an inhibitory effect (Schulte and Müller-Oehring, 2010; Turgut et al., 2023; Jäncke and Steinmetz, 2003; Josse et al., 2008; Karolis et al., 2019). Tasks that exhibit strong lateralizing activity show less commissural connectivity. In fact, one view of how hemispheric specialization occurs is based on the ability to cross-inhibit the homotopic lateralized region of the other hemisphere (Jäncke and Steinmetz, 2003).

Functional activity following focal brain injuries can further support the view that cross-inhibition is essential for maintaining higher lateralized functioning (Zhang, 2024; Cramer, 2008). Damage to areas responsible for highly lateralized cerebral functions usually reveals cross-hyperactivity in the corresponding regions on the other hemisphere. People with left-sided Broca’s area stroke show hyperactivity in the corresponding right Broca’s area. Over time, perilesional activation gradually increases, whereas right hyperactivation decreases (Bartolomeo and de Schotten, 2016; Crosson et al., 2007). The same is observed with right-hemispheric lesions in relation to spatial neglect (Corbetta et al., 2005; Corbetta and Shulman, 2011). Interestingly, these activities have been shown to be associated with functional impairment rather than compensation. A gradual decrease in hyperactivation is also related to functional recovery.

Although the mechanisms by which inhibitory interhemispheric coordination supports hemispheric specialization are less well understood at present, it appears crucial for higher-order functions, and its disruption leads to functional impairment.

Cross-inhibition can also be thought to maintain a higher level of specialization in each hemisphere on the network level. People with hemispherectomies exhibit an increasing between-networks interactions compared to healthy individuals possibly following the loss of cross-inhibitory signal in its fellow hemisphere (Kliemann et al., 2019).

#### Reality feedback

Unity of experienced consciousness is strongly driven by the unity of experienced reality, whether the physical unity of the body or the unity of the external environment. As discussed earlier, each hemisphere supports a basic level of bilateral sensory and motor functioning.

Additionally, right and left higher functions operate in an integrated manner, understanding a person’s state while recognizing their face and intentions from speech. Locating an object in an environment and reaching it by hand or identifying it by name. The right and left higher functions appear complementary rather than competitive. Synchrony in time and place is driven by reality after all.

This reality feedback is also crucial in how each hemisphere can advance the information processing progress of the other hemisphere, as discussed in part III of this article.

**Table 4:** 

Postulate 4: The conscious-mind unity is only apparent. The right and left disparate conscious experience are complementary within an actively constructed and continuously updated model “workspace” of reality.

## Part II: how the two cerebral hemispheres generate a unique view of the world

**Figure 2:**
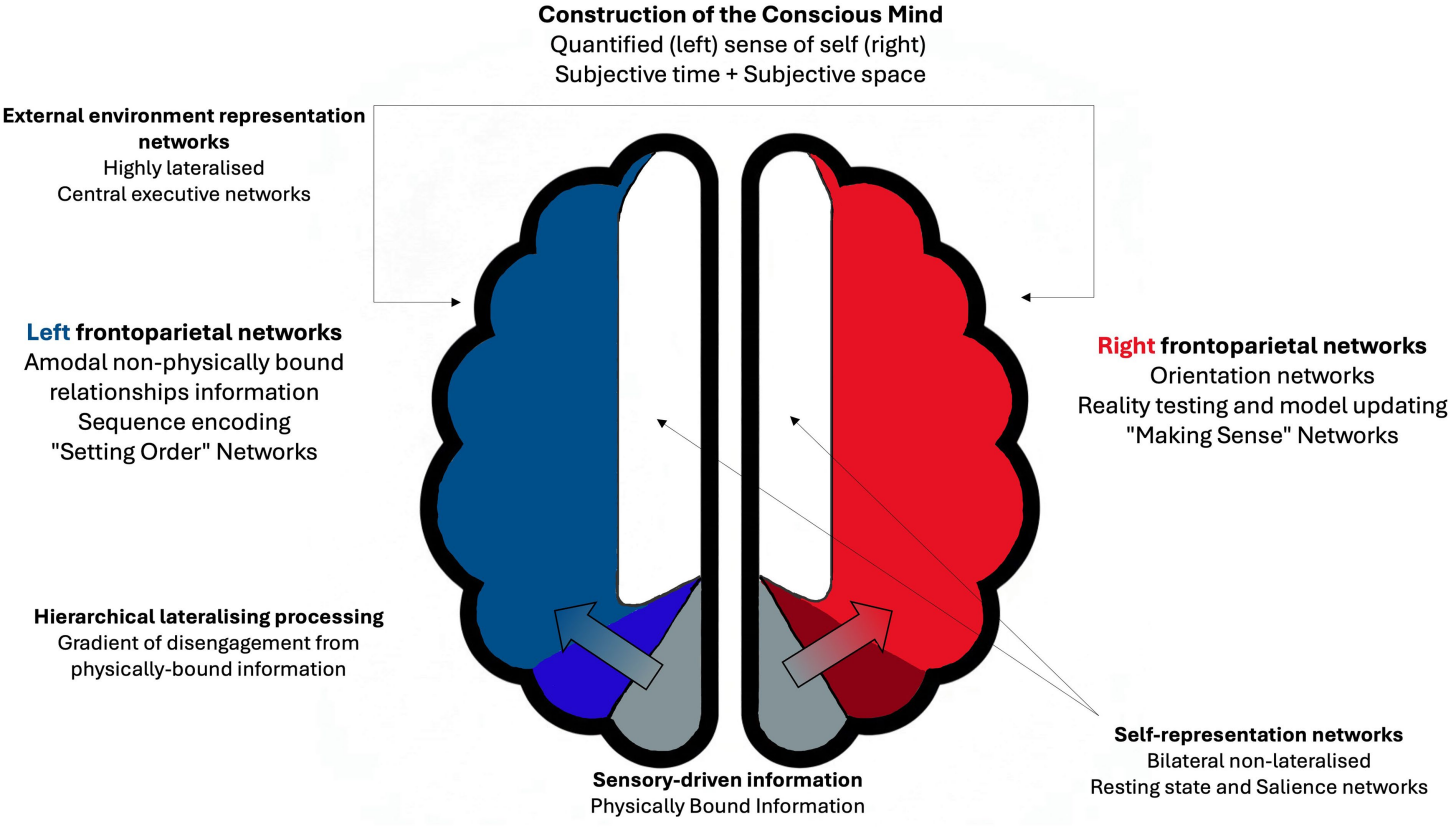
Construction of the conscious-mind. The brain maintains a bilateral representation of the self through the medial frontoparietal regions. The navigation through the external world starts with the sensory areas flowing information to the lateral parts of the brain, which is highly lateralized and independent within the human brain. Integration within each hemisphere yields a disparate sense of self, which ultimately coalesces into a unified, integral sense of self.

In this section, an outline of the information-processing disparity will be provided, beginning with 1- an overview of hierarchical information processing, followed by 2- how information-processing asymmetry emerges early in the brain, and finally, 3- how this leads to a pronounced degree of asymmetry at higher processing levels (Figure 2).

### Hierarchical information processing

The concept of Information is a purely mental phenomenon; in its Latin-derived etymology, it means the formation of the mind. It is only a mental reflection of physical reality. For our purpose, there are two classes or levels of information within the brain: a physically-bound information that is descriptive of object specifics in physical reality, in other words, sensory-driven or modal information. The second, next-level class of information concerns the relationships among different physical entities at the conceptual level (mapping patterns). This information class is abstract and amodal, not particularly restricted to any specific sensory object.

- Perceptual object information refers to the directly perceived physical entity and the defining features of a given specific object within the consciousness’s access. Seeing an apple, hearing a phoneme, or a bird sound are all examples. The information that enables the recognition and identification of an apple constitutes “perceptual object information.” This information is bound to the physical reality and the sensory experience for its existence.
- Relationship (mapping) information refers to how a given object relates to the surrounding components of the overall conscious experience. The physical, spatial, contextual, temporal, quantitative and order relationships of objects are typical examples. The order of phonemes within a word is a relationship of order (temporal mapping), the body proportions of a drawing of a person are a spatial relationship, and so on. Relationships may not only be physical but also semantic-lexical and socially contextual, and have their own extensive non-spatial–temporal mapping relationships. Relationship information can also be a mixture of the above examples; recognition of any apple is made through a combination of abstract information about size, texture, color, and even taste. This relationship information is non-immediate for any given object; in other words, it does not need to be perceptually present to have conscious access to it. You know an apple when you see one, but this knowledge is not limited only to the moment of seeing apples.
- Perceptual object information proceeds from simple to complex in a hierarchy: objects progress from simple forms, such as a single phoneme, to more complex forms, including words and even whole sentences. Relationships govern the hierarchical construction of objects, as seen in phonology and syntax. This can also be considered in the context of visual and spatial processing; a point or a line drawing is a simple object, whereas Pablo Picasso’s “Guernica” is not.
- Direct Perceptual information dissociates from its relationship-information (mapping patterns) at the higher levels of processing. Objects are implicitly encoded within their relationships without distinctions, at least during the early sensory stages. Substantive evidence indicates that spatial and temporal relationship-information (mapping patterns) is encoded within each sensory modality during the initial processing stages, rather than through a separate global system in the brain (Buonomano and Maass, 2009; Buonomano et al., 2023). In the later stages of information processing in the brain, particularly in the human brain, a distinction between perceptual object processing and relationship processing becomes clear; this will be discussed in detail later. Abstract concepts, such as order and quantity relationships, are examples of pure relationship information; they are devoid of any specific physical attributes. At the other extreme, contextual information is another example to be discussed in detail later.
- Relationship-information (mapping patterns) is strongly asymmetric. Earlier processing stages exhibit sensory domain specificity and only a small degree of functional asymmetry; later processing stages reveal wide, domain-general relationships in information processing that are not dependent on the sensory modality (amodal).

**Table 5:** 

Postulate 5: Information processing is hierarchical: lower levels are bound to consciously perceived physical objects, whereas higher levels concern relational-information (mapping patterns) that is not dependent on sensory modality (amodal) or immediate conscious access.

### Earlier information processing between the two hemispheres shows distinct asymmetry

Dissociation of information streams between the right and left hemispheres begins earlier in the processing pathways. Tonal auditory stimulations, for example, recruit the left hemisphere’s auditory cortices more preferentially (Clunies-Ross et al., 2015). Left-hemisphere auditory areas showed differential cortical columnar organization, with columns more widely spaced and wider neuropil than their right-hemisphere counterparts. This is thought to have less overlap and less redundancy in information representation (Warrier et al., 2009; Chance, 2014).

The interesting asymmetric sampling in time (AST) hypothesis posits that the left and right auditory cortices process incoming auditory stimuli utilizing distinct temporal integration windows. Specifically, this hypothesis suggests that the left auditory cortex preferentially analyses sounds within shorter temporal windows, approximately 30 milliseconds, whereas the right auditory cortex favors longer temporal windows, approximately 200 milliseconds. This asymmetry is considered essential for speech perception, where information is transmitted across multiple timescales, including rapid spectral changes and slower syllabic or prosodic information (Oderbolz et al., 2025; Poeppel, 2003).

This functional organizational pattern supports the local–global contextual mapping (relationship-building) within the right and left hemispheres. The shorter sampling time of the left hemisphere enables the formation of hierarchies with higher temporal resolution and finer granularity than those of the more redundant yet integrative counterpart regions in the right hemisphere.

The right ear (left hemisphere) advantage in verbal dichotic listening is a classical test in demonstrating hemispheric asymmetry and is classically used in functional imaging studies for such purpose (Hugdahl and Westerhausen, 2016). Such an advantage is driven through bottom-up mechanisms that involve both cortical and white matter connectivity levels (Westerhausen et al., 2009; Hickok and Poeppel, 2007).

Visual research supports a similar functional asymmetry, which begins with the earlier processing areas of the visual cortices. Simple visual stimulation showed asymmetry towards the right hemisphere (Hougaard et al., 2015). Motion perception shows earlier functional asymmetry (Chiappini et al., 2022). The right hemisphere shows equal access to both visual hemifields, in contrast to the left hemisphere, which shows greater asymmetry in reaction times across hemifields, as demonstrated by behavioral, electrophysiological, and functional imaging studies (Alberto Marzi, 2010; Putnam et al., 2010).

The spatial frequency of visual information has been suggested to be asymmetric between the two hemispheres. The right hemisphere is more attuned to low spatial frequencies, such as global features and coarse patterns, whereas the left hemisphere is more attuned to high spatial frequencies, including fine detail and edges, for example (Ivry and Robertson, 1998). This led to an interesting proposal, adapted from signal information theory, suggesting that the right and left hemispheres process sensory information at different sampling rates: a high sampling rate (left) versus a low sampling rate (right). Although not a universally consistent feature, it still provides some evidence of earlier divergence of information processing between the two hemispheres (Flevaris and Robertson, 2016; Kauffmann et al., 2014). Both cerebral hemispheres show different patterns of connectivity; the left hemisphere exhibits more intrahemispheric connections, while the right hemisphere’s connections span across the two hemispheres through interhemispheric connections (Gotts et al., 2013). Shorter intrahemispheric connections permit high spatial and temporal resolutions, while the integrative connections of the right hemisphere can capture the lower spatial resolution. This difference in perceptual frequency has also been suggested to correlate with motor action patterns, and, more specifically, with handedness (Raymond et al., 1996; Morgan and Casasanto, 2025).

**Table 6:** 

Postulate 6: Information processing asymmetry begins early on each side of the brain, driven by key structural differences at both the cortical and white-matter connectivity levels. This asymmetry mediates different mapping capabilities between the two hemispheres.

### Advanced processing of relationships

In contrast to earlier perceptual levels of processing, the more advanced stages, particularly the frontoparietal networks, exhibit strong and consistent lateralization. Higher levels of processing further augment the earlier asymmetry in sensory processing.

Based on the observed frequency difference, high-frequency sampling in the left hemisphere provides high-resolution details to the immediate local relationships between neighboring components. In contrast, lower-frequency sampling in the right hemisphere is better at recognizing more distant, global relationships.

Local relationships are fundamental in the construction of a word from phonemes and of a sentence from words, where the immediate sequence of objects (phonemes or words) is crucial. Global relationships are more effective in establishing intricate connections within a perceived visual scene, creating a unifying context for the multiple diverse components.

The following will outline how, at higher-level processing, the left hemisphere establishes order relationships; high-resolution, low-dimensional relationships (temporal/quantitative mapping), while the right hemisphere forms multidimensional, lower-resolution integrative contextual relationships (spatial/contextual mapping).

#### Setting order

What is common to language, logic, quantification, and the sense of time is their strong dependence on order and on the rules that govern it. Logic relies on a strict, unidirectional temporal order in which cause precedes effect, whereas language exhibits more complex order relations, including linguistics, phonology, morphology, and syntax. Order is the result of the combination of granularity and time directionality. In a sentence, each word occupies a position, and the meaning is determined by reading the sentence in one direction rather than the other.

Granularity can be achieved by establishing high-resolution, precise boundaries between objects. This characteristic originates early in the left hemisphere’s sensory processing, providing a discrete quality within the context. Phonemes (discrete) order generates a word (object made through ordered components), words in order (discrete) create a sentence (object made through ordered components) and so on. Cause precedes effect; 10 is larger than five; order begets order.

Several neural mechanisms have been implicated in establishing such granularity and determining the direction of order. These mechanisms range from simple to highly complex and involve multiple brain regions. Contemporary statistical learning research proposes two systems that operate to support such sequential learning abilities (Dehaene et al., 2015; Conway, 2020). Learning simple sequences is relatively easy for the nervous system and can be considered an inherent implicit property of the nervous tissue. These processes are believed to operate in different brain areas in a modality-specific manner: visual sequences are learned in early visual processing areas, whereas auditory sequences are learned in auditory regions (Conway, 2020).

Some mechanisms mediating this sequence learning are shared with many other animals, such as transitions and timing knowledge (when should one item stop and the next item start) (Balci et al., 2009; Kheifets and Gallistel, 2012), chunking (grouping contiguous, similar items together) (Bekinschtein et al., 2009; Basirat et al., 2014; Uhrig et al., 2014), and ordinal knowledge (which item comes first and which comes next, regardless of gaps) (Chen et al., 1997; Nieder, 2012).

While transitions and timing knowledge, as well as chunking, are implicitly automatic and may not even require attention, ordinal knowledge constitutes a form of abstraction. It represents an independent analysis of relationships (Dehaene et al., 2015). More complex forms of sequences, however, involve another higher system that is strongly lateralized towards the left frontoparietal networks. Interestingly, similar but less specialized networks are shared with other primates (Milne et al., 2018; Wang et al., 2015). More complex and advanced sequence coding, such as algebraic patterns (regularities within a sequence) (Wang et al., 2015; Marcus et al., 1999) and nested tree structures (complex non-flat sequences, much involved in language syntax) (Chomsky, 1956; Chomsky, 2013), is well-observed only within the human brain and strongly left-lateralized within the frontoparietal networks (Brennan et al., 2012; Pallier et al., 2011).

The left inferior frontal region appears to be responsible for such ordering within a universal domain-general network, regardless of the sensory modality involved (Conway, 2020; Milne et al., 2018; Thothathiri and Rattinger, 2015; Tettamanti and Weniger, 2006; Wilson et al., 2017; Hammond, 1982). The universal non-domain-specific nature of such a network also implies that it is not “object-specific” but rather mapping-pattern-specific and “relationship-specific.”

Quantity estimation is known to relate to the left hemisphere and can be thought of as a simultaneous, discrete representation of the granularity of the number of objects being considered.

**Table 7:** 

Postulate 7: The left hemisphere’s information processing hierarchy mediates order and quantity mapping patterns or relationships. At higher levels, these types of information are strongly lateralized to the left frontoparietal networks.

#### Making sense

Abstraction is the process of extracting rules from a context, etymologically driven from the verb abstrahere, meaning “draw away.” The opposite is contextualization, from Latin contextus, from con- “together” + texere “to weave.” “Making sense” is the mental process of integrating new information with existing knowledge or mental models to make it meaningful. It can be seen as a counteraction to abstraction.

Abstraction involves discovering rules regardless of the available components at hand, whereas making sense involves integrating the present components irrespective of the pre-existing rules.

From that perspective, the right hemisphere’s hierarchical processes are in opposition to those of the left. It is highly context- and component-bound. Recognition and discrimination, reliant on the right hemisphere, function by assimilating and accommodating information into learned models. Interestingly, patients with right-hemisphere damage and split-brain patients struggle with these functions, despite the analytical processes of the left hemisphere.

This context-synthesis within the right hemisphere is crucial in map-based perceptual systems, such as visual and somatosensory systems. In the retinotopic map, points and lines are represented in V1/V2. At higher levels, patterns are identified regardless of their location or basic map features. This is “perceptual invariance,” allowing recognition of objects, regardless of size, orientation, brightness, or other visual properties. It enables us to recognize a face or an apple, whether they fill our visual field, are in a close-up, or are several meters away.

Perceptual invariance is an automatic, unconscious, form of statistical learning exhibited throughout the nervous tissue of most animals (Conway, 2020; Cichy et al., 2014). As will be highlighted in part III, artificial neural networks have successfully replicated this encoding hierarchy in Convolutional neural network models (CNN), where multilayered feature-extracting filters capture visual patterns, such as geometric shapes or faces, within a photo (Dehaene, 2021; LeCun and Bengio, 1998; LeCun et al., 2010).

At a further higher level, this context synthesis goes beyond object recognition. It extends to what is expected from this object’s behavior, something that will be referred to here as “Agent recognition.”

Within the agent recognition concept, objects, including animals and human beings, are recognized in a dynamic context; they are perceived within a dedicated contextual spatiotemporal map. Physical laws exemplify context-mapping of inanimate objects in motion, and intentionality of others is perceived within another context-mapping about human beings (Theory of the Mind) (Corbetta et al., 2008; Corbetta et al., 1993; Baldauf and Desimone, 2014; Arrington et al., 2000; Barnes et al., 2001).

Inanimate objects have numerous contexts and expectation mapping beyond physics. We expect a tree to have apples or other fruits hanging, but not eggs, for example. We expect apples and other objects to be within a specific size range, not the size of a grain or the size of a van, for example. These innumerable contexts are commonly attributed as “common sense”; the right hemisphere operates the neural processing of making sense. Agents (objects and human beings) make sense when they are recognized within their context, expectation mapping.

These contexts are modulated within the brain, and the right ventral frontoparietal network is consistently activated for updating such models whenever a reorienting bottom-up input does not align with the expectation (Corbetta et al., 2008). Violation of expectation always recruits the domain-general right hemisphere resources, while the left hemisphere is involved if such violations involve some of the left hemisphere-specific processes, such as verbal or abstract physical laws (Liu et al., 2024; Cloutier et al., 2011; Bubic et al., 2009). These circuits are key to reality testing and comprehension, discussed earlier in the context of delusional disorders of split-brain and right hemisphere damage patients.

It is the same regional network, the inferior frontoparietal network, that is activated whether a car suddenly appears on the road, when a cup falls out of the table edge, and when a person says hello and looks into your eyes (Van Veluw and Chance, 2014; Mitchell, 2008; Adolphs, 2009). There is a topographical distinction in activation across contexts, such as social (Theory of the Mind) versus non-social (common sense). However, the proximity of these different activations within the posterior temporoparietal areas and the inferior frontal regions is unmistakable.

If an apple defied the laws of physics and flew up instead of being pulled down by gravity, this would have activated the right ventral attention network in Newton’s brain. This attentional network is activated whenever a model update is needed because the current model did not expect the event to happen.

Developmental and behavioral psychology also reflects this universal “Agent recognition” concept and how it develops in our minds. Subjects are “objects that carry self-attributes,” and physical objects can be perceived with animalistic qualities. Understanding these highly complex behaving objects, “animals, particularly human beings,” their intentions, and expected behavior is central to object modeling within the right inferior frontoparietal network. It provides another way of ascribing meaning to objects—alive, as I am, and even part of me. It is interesting to see how Jean Piaget described the early cognitive pre-operational stage. According to Piaget, the child begins with a complete fusion between the self and the world (reality). Then, a gradual process of separation between the two occurs—internalization of the self and externalization of the world. This process is never fully complete, even in adults (Piaget, 2013; Piaget, 2017).

Analyzing and synthesizing context is a style of integrative information processing and allows us to see fluidity and continuity in what is separate and discrete. Different body parts make up one human being, an integrated, unified object that is made up of various different components that “make sense” together. This integrative encoding is in remarkable contrast to the abstraction and granularity encoding of the left hemisphere.

It is interesting that the analogy of how artificial neural networks, which model language learning, aren’t ideal for image analysis. In contrast, convolutional networks, which excel at image analysis, fail at language learning. These processes are widely separated in the brain for reasons related to neural information-processing levels.

**Table 8:** 

Postulate 8: The right hemisphere’s information processing hierarchy mediates complex contextual and integrative mapping patterns or relationships. At the higher levels, networks that maintain and update these complex mental models are strongly lateralized to the right hemisphere.

## Part III: hemispheric disparity in action

### Explaining disparate hemispheric processing through mathematics

Within the hemispheric disparity view, conscious-mind engagement with reality generates separate mental/cognitive concepts being created independently in each hemisphere, driven by the same experienced objects. While these two disparate pairs of concepts act upon the same reality target, they never coalesce into a single whole, and we switch between right- and left-driven concepts from one time to another. For example, visual recognition and naming are strongly lateralized, separate functions that operate simultaneously. At a given moment, one of these lateralized functions can predominantly utilize cognitive resources and dominate the engaged conscious-mind process. Hemispheric disparity posits that this independent process with alternating dominance is a crucial step in the development of thoughts, concepts, and even culture.

It is important to note that we always use the two disparate hemispheres together at all times and across all experiences; however, at any given moment, one process tends to utilize cognitive resources predominantly, being perceptual or executive. Empirical observations show bilateral activation in most cognitive tasks, with selective lateralized activation occurring only when the testing task is controlled and repeated sampling is used.

Rethinking some of the following mathematical concepts can serve as a thought experiment demonstrating such a hemispheric disparity process and also provide a good model for developing experimental tests of the theory.

#### Discrete and continuous concepts in geometry

Let us start with the concept of geometrical space. Within such a space, let us consider the “geometrical point,” the most fundamental concept in geometry. It is defined as: an exact location in space with no length, width, depth, or dimension.

Specifying a “point” in “space” with defining rules makes the point concept a discrete concept, seen in isolation from its context, and it is self-identifying without reference. Discrete concepts can be described within a set of defined features; in other words, you can define each concept.

Let us now take a moment to think about how this point is hypothetical and abstract; there is nothing in reality that can materialize all these properties. Materializing this point in real space would make it occupy a length, width, and depth, which would violate its core defining properties, namely, having no length, width, or depth.

Our ability to understand and quantify a discrete point in space is a left-hemispheric process; it involves a concept (having a definition or a category) and a quantity (one point), and these two processes are known to lateralize to the left hemisphere.

What is interesting here is that a hypothetical and abstract concept can be reflected in reality without actually being part of it: “a lie that makes us realize truth,” as Pablo Picasso put it (Gilot and Lake, 1964).

We can hold a pen and draw a single point on paper. By viewing this point, we engage the right hemisphere object/agent recognition and materialize a context for such a point. This context enables us to engage with this point, judge its significance and how to utilize it once it is on paper.

According to the hemispheric disparity theory, two highly intriguing mental processes occur in this context

1. The human brain is capable of holding both a contextual, realistic, and an abstract, hypothetical view on this very point in an independent and simultaneous fashion between the right and the left hemispheres.
2. The human brain can allocate conscious behavior or cognitive-control resources between the two views in a progressive, recursive fashion.

Once a perceived object materialized in reality (right), further hypothetical rules can be added on (left). For example, we can imagine further (utilizing the left hemisphere’s predictive capacity) and make assumptions about quantity or order to support our point, execute them, and see how it works in reality (right hemisphere object-context update).

Adding another point on the paper next to the first creates a reality of two discrete points on the paper. These two points can be visualized as connected by a continuous line (using the right hemisphere’s predictive capacity). This line can be further analyzed as an infinite number of points, starting and ending at our two points on the paper (left-hemisphere abstraction).

This is a mutual/alternating cycle of materializing and hypothesizing. Adding the next hypothetical concept was made possible or easier after materializing the previous one, the hypothetical “point concept.” This is a counterfactual process in action, a feature that distinguishes human from machine learning, as reported next (Pearl and Mackenzie, 2018).

Here, the left abstract processes progressed further thanks to the support of the right hemisphere. This is an interdependent process between the left and the right.

This mutual recursion can proceed endlessly, from a line to a 2-D shape, a 3-D shape, or any other set of abstract rules (weight, surface, order, quantity, etc.) that we can overlay on observed reality in the same recursive way described above.

Notably, at each step forward, reality is perceived in a completely different way once an abstract concept is overlaid upon it. From an object recognition point of view, a point is not a line, a line is not a square, and a square is not a cube. Additionally, at each step forward, abstract concepts become more complex and embedded. Rules that define a 3-D shape are more complex than those defining a 2-D shape, such as a line or a point.

These two mental processes are the visual counterparts of the two key features of human language: symbolism (the mutual attribution of abstract and hypothetical to realistic concepts) and recursion. Hemispheric disparity processing gave rise to both language and the mind.

There is supporting evidence for these right–left–right transformations within the brain. Transforming order to insight is a left-to-right transformation strategy. In the classical remote association tests, left-hemispheric activity matches the search strategy until the moment of insight, when sudden right-hemispheric activity ensues. This is known in literature as the “Aha! moment,” and it shows a strong lateralization of the right temporal lobe in fMRI and EEG studies (Kounios and Beeman, 2009; Dietrich and Kanso, 2010). The Aha moment is vivid, but the same processes occur at smaller scales and are applied continuously with every update to the perceptual reality model.

On the other hand, right-to-left transformation occurs during experimental engagement with right-hemisphere reality models through left-hemispheric control networks. Directing overt attention, grasping, and naming are all direct examples of such right-to-left transformation. Overt attention can be understood as an action that updates perceptual models (Parr and Friston, 2019; Friston et al., 2012). Face-name associative learning depends on the right hemisphere for newly learned faces and names, as well as the lexical resources of the left hemisphere, as demonstrated by lesion and fMRI studies (Tsukiura et al., 2002).

**Figure 3:**
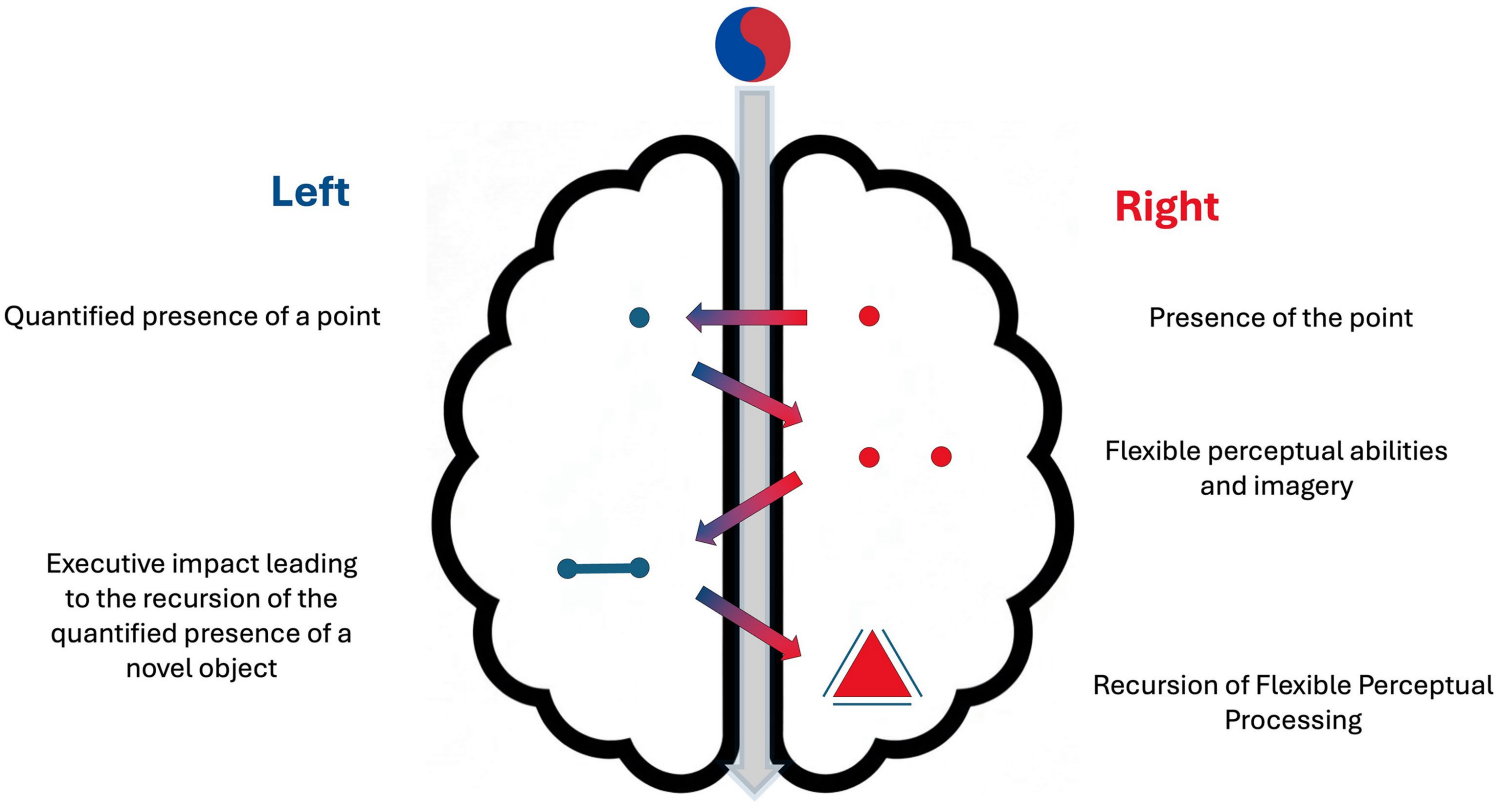
How the disparate hemispheric processing generates progressive recursion that mediates concept development. Each hemisphere’s processing outcome serves as a feed-forward to a parallel process in the other hemisphere.

Future experimental designs based on similar geometrical or mathematical analogies can address these transformations and provide insight into how hemispheric laterality operates in real life (Figure 3).

**Table 9:** 

Postulate 9: Through the two disparate hemispheric processes, the conscious-mind can hold, simultaneously, the analytical and rule-extracting left hemisphere and the context-synthesizing right hemisphere. These two processes can be recursive, one feeds to the other in tandem, allowing for concept building and thought progression.

#### Descartes’ precision: analytical geometry, calculus, and more

The ability to transform what is essentially spatial (a graph) to what is essentially quantifiable, and verbal (an equation) is a right-to-left transformation. Within mathematics, we were able to solve visually unsolvable problems using verbal analytical mechanisms, thanks to the laws of analytical geometry. Likewise, we can feed quantifiable values through a set of variables to generate a visual representation using curves in coordinate space.

Isaac Newton and Gottfried Wilhelm Leibniz’s work on the “area under the curve” is another example of the transformative power of cross-manipulating spatial phenomena into quantifiable and verbal forms. The Fourier transformation is a third example among many others. A Fourier transform is a mathematical process that breaks down a complex function into a sum of simple sinusoidal (sine and cosine) waves of different frequencies. It converts a signal from its original domain, such as time or space, to a new domain that represents its frequency components. This allows us to analyze and manipulate signals by understanding their constituent frequencies, which is essential in fields like image processing, signal processing, chemistry, and physics.

The analogy with signal information theory is contemplative. The ability to digitalize analog signals (transforming continuous analog signals into discrete chunks of information) has been revolutionizing communication in the modern world.

Rene Descartes, the renowned philosopher and mathematician, along with Pierre de Fermat, contributed to the development of analytical geometry, which, in this article’s view, relies on the same core process that generates our conscious experience.

Descartes’s philosophical views on consciousness sparked influential debate on the nature of human consciousness as he proposed the concept of duality, separating what is mental from what is physical. This proposition has been consistently rejected in the modern literature on the philosophy of the mind and consciousness. Antonio Damasio’s book titled “Descartes’s Error” is highly reflective on this fact (Damasio, 2006). Ironically, “Descartes’s precision” in mathematics should now even out his philosophical error.

## Hemispheric disparity—where did it come from, and where can it lead us to?

### Where did the conscious-mind come from?

It is difficult to answer this challenging question, if at all; however, there is an interesting assumption regarding a sequence of events. It is probably the progressive increase in brain size that created relative functional independence between the two hemispheres, which, in itself, allowed for this extreme form of functional asymmetry and a parallel disparate conscious experience characteristic of the human mind.

Primate brains show a progressive increase in size in this energy-demanding tissue across evolutionary time; human brains obviously reached the pinnacle of this relative increase in size. Whether the driving force has been an abundance of energy resources, improved energy utilization, or a key cognitive or social adaptation that expanded access to such energy, size did matter in this story (Aiello and Wheeler, 1995; Isler and van Schaik, 2006; Navarrete et al., 2011).

From a phylogenetic perspective, as the nervous tissue space increases in size, the biological cost of maintaining the same level of connectivity becomes prohibitively high. As size increases, regions operate with relative independence, supported by increased local connectivity within each region and decreased high-throughput connections between distant areas. This networking style, “small world networking,” has been consistently observed across the evolving vertebrate brain, with the common theme of “larger equals disconnected” (Striedter, 2005).

While the general layout of the cerebral cortex is similar across primates, some areas, particularly high-level association regions such as the temporal parietal junction and inferolateral prefrontal cortex, exhibit disproportionate enlargement in larger brains, with the most significant expansion occurring in the human brain (Chaplin et al., 2013). These are the same areas involved in highly lateralized regions in each hemisphere that set order and make sense in our minds (Patel et al., 2015; Doricchi et al., 2022).

Despite the brain size expanding in humans, the main connecting bundle between the two hemispheres, the corpus callosum, has relatively shrunk in size, even compared to primates, implying that hemispheric independence is a purposeful key feature, particularly within highly processed, lateralized functions (Phillips et al., 2015; Hopkins and Cantalupo, 2008; Rilling and Insel, 1999; Hopkins and Rilling, 2000; Hopkins et al., 2015).

The larger association areas show smaller and slower commissural fibers within the corpus callosum compared to the lower-level sensorimotor processing areas. One viewpoint is that the conduction delays in large brains across the two hemispheres are longer than they should be to facilitate complex, time-sensitive computations, which has resulted in the adaptive specialization of local, independent, lateralized networks (Turgut et al., 2023; Ringo et al., 1994).

As discussed in Part II, independence produced distinct information-processing styles in each hemisphere. It has always been thought that hemispheric specialization is an energy-saving strategy to avoid a lengthy, time-consuming travel of information between the two hemispheres. But as hemispheric disparity proposes, independence is needed for isolating different information coding styles and ultimately parallel experience. Mixing these two different information coding would collapse this unique conscious-mind design. Lateralization is essential in mental and social development on many scales, and various developmental and neuropsychiatric disorders show significant alteration of hemispheric specialization, such as autistic spectrum disorder and schizophrenia (Philip et al., 2012; Oertel-Knöchel and Linden, 2011). Hemispheric specialization is also linked to creativity, and functional divergence between the two cerebral hemispheres contributes to human intelligence (Liang et al., 2025; Mihov et al., 2010).

This purposeful isolation of processing between the two hemispheres also applies on the behavioral outcome level. As each hemisphere is able to influence behavior independently, one hemisphere’s behavioral outcome influences/updates its fellow hemisphere’s reality model in a novel way. As suggested earlier, hemispheric disparity theory posits that tandem processing is crucial for concept development and accumulation.

**Table 10:** 

Postulate 10: Hemispheric specialisation is not merely an energy conservation strategy. The large, structurally separate hemispheres allow for two separate information coding styles to develop, each in its own hemispheric hierarchy. Lack of such separation would hinder the hierarchical development of each information coding style and ultimately the disparate conscious-mind process.

### Where can hemispheric disparity lead us?

There are numerous potentially important applications of hemispheric disparity across philosophy, the clinical sciences, and artificial intelligence.

#### The emergence of mind and time

The lateralized higher functions in each hemisphere rely on the basic processing level in the corresponding hemisphere, generating an overlapping sense of self. One that is able to synthesize a dynamic sense of self across the order of time that is lateralized to the left hemisphere, and an integrative overlaying observing self is lateralized to the right hemisphere.

The integrative, context-rich processing of the right hemisphere creates a coherent self in continuity with the outside world and unifies the two worlds (outside and inside) as a cohesive whole. The right hemisphere drives the integrated sense of presence in the moment.

On the other hand, the relationship information in the left hemisphere creates a discrete, quantified sense of self that is dynamically presented over time. Autobiographical episodic memory is left prefrontal cortex dependent in comparison to non-autobiographical episodic memory (Gilboa, 2004). The autonoetic self and the autonoetic episodic memory are left-hemisphere-driven concepts, based on the abstract notion of time.

The type of episodic memory that is aligned across the timeline is well pronounced in humans. It should be distinguished from the event recall, which is widely shared and demonstrated across mammals and other species (Burgess et al., 2002; Allen and Fortin, 2013). That former type of episodic memory is the latest to develop in our mind, the earliest to be lost in Alzheimer’s disease and related disorders, and the autonoetic form of this episodic memory is claimed to be what distinguishes the human mind, according to Endel Tulving (Wheeler et al., 1997; Nyberg et al., 2010).

Our dynamic perception of time—the dynamic theory of time—is a process that can be traced within the human brain. Our conception of time directionality, traveling from the past to the future through the present moment, is based on the referential autonoetic self in the here and now. This episodic nature of time is a quantified abstract representation of events and actions, which can be seen as a left-hemispheric-driven function. Combined with the integrated sense of self from the right hemisphere, a timeline can be constructed.

In philosophy and modern physics, there is a growing acceptance of the static theory of time, which holds that the flow (motion and directionality) of time is not a genuine property of the universe (Rovelli, 2019; Baron and Miller, 2018). The construction of time within the conscious-mind can be traced in the brain, and it appears to be woven into the fabric of our conscious experience, making a static theory of time quite challenging to digest, despite the strong, well-established physical evidence available. It does not seem logical because logic itself is dependent on the same dynamicity we understand time within; events lead to others through a cause-and-effect framework.

Within the hemispheric disparity theory, the concept of time as we dynamically experience it is a constructed model within the fabric of the conscious-mind, even when it appears not to be within the fabric of the universe. There is no meaning in separating the sense of time from the experiencing self, at both the cognitive and physical levels. Time is a relative, observer-dependent construct.

#### Clinical models of laterality

Certain clinical disorders, including autistic spectrum disorder, schizophrenia, and dyslexia, are linked to altered cerebral lateralization (Ocklenburg et al., 2024; Packheiser et al., 2025). Hemispheric disparity provides a useful model for understanding changes in laterality across various neurological and neuropsychiatric conditions. In this context, it is plausible that some disorders primarily involve altered lateralization. Currently, bedside assessment of laterality is limited. It remains unclear how variations in the side of lateralization (right versus left) and magnitude (strong versus weak), for functions like language, spatial attention, facial recognition, and emotional processing, influence the balance or imbalance within the brain’s overall structure. Differences in the presentations of disorders such as Alzheimer’s, Parkinson’s, or other neurodegenerative and neurodevelopmental diseases can be analyzed through hemispheric disparity as a factor shaping cognitive and mental functions (Steinbach et al., 2023; Abuduaini et al., 2024).

Furthermore, this model may enhance therapeutic and rehab strategies by focusing on supporting specific functions in one hemisphere via targeted training or stimulation, or on reducing interference from the opposite hemisphere (Desarkar et al., 2015).

#### Artificial intelligence

### Hemispheric disparity is a state of a continuously updated reinforcement learning model

Artificial intelligence learning models can be classified as supervised or unsupervised. An interesting analogy to the dual-conscious processing of the human brain is the type of supervised machine learning known as “reinforcement learning” (Dehaene, 2021). In this type, a final goal is specified for the learning algorithm, and two models compete to achieve it more effectively. The successful model between the two is adopted and updated within the system; the process is then repeated, with such improvements at each step. It is tempting to speculate that right- and left-hemisphere-driven processes may compete for control resources, with the final conscious behavior determined by success in approaching value-based goals and outcomes. In the next step, the behavioral outcome and environmental change trigger a model update for the next run, and so on.

One important caveat in the analogy with reinforcement learning is that, in the human case of disparate hemispheric processing, each hemisphere’s outcome serves as new data fed to the other hemisphere. This creates a dynamic, potentially infinite process driven by environmental input. In contrast, machine learning relies on a finite dataset and lacks the generative, self-feeding conscious experience of the human brain. This is human creativity, put differently. The human brain is equipped with a continuously updated and modeled set of ultimate goals and targets, unlike AI models that have predetermined, fixed goal outcomes.

### The convergent evolution of AI models

One notable observation is that artificial learning models must rely on two distinct algorithms for image and language processing. As outlined in part II, convolutional neural networks (CNNs) have demonstrated strong performance in image and contextual analysis across multiple fields (LeCun and Bengio, 1998; LeCun et al., 2010). On the other hand, large language models (LLMs) have been revolutionary in the analysis and processing of language information (Vaswani et al., 2017). Still, each type of algorithm performs better in its corresponding domain, and NNs still outperform LLMs when it comes to image and complex contextual analyses (Dosovitskiy, 2020; Gupta et al., 2025; Liu et al., 2025).

Hybrid multimodal AI is emerging, and Modern Vision-Language Models (VLMs), such as GPT-4o and Gemini, often employ a hybrid approach. According to the hemispheric disparity theory, a similar hybrid approach has been employed in the modern human brain over the last 300 thousand years.

Understanding how the human conscious-mind is linked to learning, as illustrated in the hemispheric disparity model, is advancing the potential for further development of artificial intelligence strategies. Adopting self-learning strategies that auto-feed information across disparate processes, analogous to the two cerebral hemispheres, can be a way forward for advancing artificial learning models.

## Data Availability

The original contributions presented in the study are included in the article/supplementary material, further inquiries can be directed to the corresponding author.
